# Increasing organ donation via anticipated regret (INORDAR): protocol for a randomised controlled trial

**DOI:** 10.1186/1471-2458-12-169

**Published:** 2012-03-08

**Authors:** Ronan E O'Carroll, Eamonn Ferguson, Peter C Hayes, Lee Shepherd

**Affiliations:** 1 Psychology, School of Natural Sciences, University of Stirling, Stirling, UK; 2 School of Psychology, University of Nottingham, Nottingham, UK; 3 Department of Hepatology, University of Edinburgh, Edinburgh, UK; 4 Scottish Liver Transplant Unit, Edinburgh Royal Infirmary, Edinburgh, UK

## Abstract

**Background:**

Throughout the world there is an insufficient supply of donor organs to meet the demand for organ transplantations. This paper presents a protocol for a randomised controlled trial, testing whether a simple, theory-based anticipated regret manipulation leads to a significant increase in posthumous organ donor registrations.

**Methods:**

We will use a between-groups, prospective randomised controlled design. A random sample of 14,520 members of the adult Scottish general public will be contacted via post. These participants will be randomly allocated into 1 of the 4 conditions. The no questionnaire control (NQC) group will simply receive a letter and donor registration form. The questionnaire control (QC) arm will receive a questionnaire measuring their emotions and non-cognitive affective attitudes towards organ donation. The theory of planned behavior (TPB) group will complete the emotions and affective attitudes questionnaire plus additional items assessing their cognitive attitudes towards organ donation, perceived control over registration and how they think significant others view this action. Finally, the anticipated regret (AR) group will complete the same indices as the TPB group, plus two additional anticipated regret items. These items will assess the extent to which the participant anticipates regret for not registering as an organ donor in the near future. The outcome variable will be NHS Blood and Transplant verified registrations as an organ donor within 6 months of receiving our postal intervention.

**Discussion:**

This study will assess whether simply asking people to reflect on the extent to which they may anticipate regret for not registering as an organ donor increases organ donor registration 6 months later. If successful, this simple and easy to administer theory-based intervention has the potential to save lives and money for the NHS by reducing the number of people receiving treatments such as dialysis. This intervention may also be incorporated into future organ donor campaigns.

**Trial registration number:**

ISRCTN: ISRCTN92204897

## Background

There is an insufficient supply of donor organs to meet the demand for organ transplantations worldwide. Over 10,000 UK residents are currently on the waiting list for a solid organ transplant, and 3 patients die per day before they receive a transplant [1]. A recent survey found that 90% of the UK general public approve of organ donation [2]. Despite this, only 30% of people in the UK (38% in Scotland) have registered as posthumous organ donors. This discrepancy suggests that there are important barriers that deter people from registering as an organ donor. The UK Government has set aspirational targets for increasing numbers on the Organ Donor Register from the current level of 17 million to 25 million by 2013, and to increase the number of organs donated by 73% [2]. There is therefore an urgent need to identify factors that promote and overcome the barriers that deter people from registering as an organ donor [3].

Traditional social-cognitive models postulate that actions are determined by rational-cognitive factors. For example, the theory of planned behaviour (TPB) [4] suggests that actions are determined by rational cognitive attitudes, subjective norms and perceived control. According to this theoretical model, people are likely to register as posthumous organ donors when they have a positive attitude towards donation ('organ donation is a benefit to humanity'), think that significant others support registration ('my family and friends think that I should register as an organ donor'), and believe that they have the ability to register ('it is easy for me to register as an organ donor'). Indeed, most behaviour change interventions adopt such social cognitive approaches. Information is given that is designed to change people's thoughts and perceptions of a specific health behaviour. For example, in 2009 the Gift of Life campaign used the slogan "Be a hero, put the kettle on", which aimed to increase organ donation by emphasising that registration was quick and easy, thus trying to alter people's perceived control over this action. Moreover, this slogan also associated organ donation with positive perceptions ('being a hero'), thereby increasing people's positive attitude towards this action. According to the TPB, such campaigns are likely to be effective. However, without rigorous scientific evaluation, it is difficult to determine their effectiveness.

Although the TPB has been found to predict a variety of actions [5,6], including organ donation [7,8], it has been criticised for not accounting for the affective/emotional factors that guide decision making [9]. Indeed, recent research in the US [10] and the UK [11,12] has found that TPB variables are weaker predictors of organ donor registration in comparison to people's emotions and non-cognitive affective attitudes towards registration, suggesting that the key to increasing organ donor registration is to target these emotions and affective attitudes, rather than traditional social-cognitive factors. These emotions and affective attitudes can include feeling disgust towards the idea of organ donation (the "ick factor"), the superstitious belief that registration will in some way lead to harm or death for the registrant (the "jinx factor"), the desire to keep the body whole after death (bodily integrity), the fear that doctors may hasten the death of seriously ill patients in order to harvest their organs (medical mistrust), and the positive consequences of organ donation (perceived benefits). Non-donors are more likely than donors to feel these emotions and hold these negative affective attitudes, and are less likely to endorse the perceived benefits associated with organ donation [10,12]. It should be noted, however, that due to the cross-sectional design of the latter studies a causal relationship cannot be established between the affective attitudes and organ donor registration. In the present study we will address this issue by using a prospective RCT design. We also aim to extend this line of work by investigating other emotions that may affect the decision to register as an organ donor or not. Our specific focus is on the emotion of regret.

### Regret

Regret is an aversive counterfactual emotion that is experienced when people believe that their current situation could have been better if they had acted differently [13,14]. It is also possible to *anticipate *the amount of regret that one would feel for undertaking or failing to perform an action, thereby giving people a pre-emptive strategy for avoiding this aversive emotion [15,16]. The desire to avoid this emotion motivates people to undertake (or avoid) actions when they anticipate feeling regret for inaction (or action). Anticipated regret, therefore, binds people to an action by signaling the aversive emotional consequences of inaction. Indeed, research from a variety of disciplines, ranging from psychology to economics, has found that anticipated regret influences decision making and the likelihood of an action being undertaken [15-21]. Anticipated regret has also been found to predict people's intentions to perform an action and actual behavior over and above the traditional TPB components [22,23]. For example, this has been found for driving behavior [20,24,25], condom use [26-30], exercising [31,32], and weight loss [33].

A growing body of research has assessed the effectiveness of using anticipated regret in behavior change interventions. Richard and colleagues [34] found that asking students to rate how they would feel after undertaking unsafe sex increased self-reported condom use 5 months later. Although this finding is promising, it could be criticised for focusing on self-reported measures. A stronger test of the effectiveness of anticipated regret in behavior change interventions was conducted by Sandberg and Conner [18]. They invited three groups of women for cervical screening: a control group, a group sent a TPB questionnaire and a group who were sent a TPB questionnaire plus anticipated regret questions. For those that completed and returned the questionnaire attendance rates were 21%, 44% and 65% respectively. This is a quite remarkable "mere measurement" effect, given the simplicity of the intervention. Simply asking people to think about and rate the amount of regret that they anticipated for not attending a cervical screening dramatically increased screening attendance.

Similar results have been found for research with more direct relevance to organ donation. Godin and colleagues [35] randomly assigned 4,672 participants to an experimental condition that received a postal questionnaire measuring cognitions about blood donation (including anticipated regret items) or a control group that did not receive a questionnaire. Compared to control participants, the mean frequency of number of registrations at blood drives among participants in the experimental group was 8.6% greater at 6 months, and was 6.4% greater at 12 months. Significant effects were also observed for successful blood donations at 6 months and 12 months. Recently, Godin and colleagues [36] conducted a further randomised controlled trial which attempted to increase blood donation in 4,391 novice donors. They found that; (a) questionnaire completion led to a significant increase in donations, and (b) simple "if-then" planning, specifying how, where and when donation would occur (implementation intentions) led to a 12% increase in donations. Manipulating anticipated regret in this study did not augment the intervention effect. However, this study (unlike others) measured anticipated regret with isolated questions, and the authors speculated that this may have been too blatant and that participants may have interpreted the obvious anticipated regret questions as an unsubtle emotional appeal. Godin and colleagues [36] also suggest that the level of anticipated regret may need to be substantial for it to change intentions and behaviour, and failing to donate blood may not engender sufficient feelings of regret. Godin and colleagues [36] conclude "Further research is needed concerning the blatancy of the induction of anticipated regret and the role of underlying levels of anticipated regret in explaining the behavioural impact of this type of intervention" (p. 643).

The research cited above suggests that subtle anticipated regret interventions increase the likelihood of an action being undertaken. Subtly increasing the prominence of anticipated regret in the decision making process emphasises the aversive emotional consequences of inaction. The desire to avoid the aversive feeling of regret motivates people to undertake the behaviour. Essentially, anticipated regret strengthens behavioural intentions and binds the person to action, because failing to act is associated with aversive emotions. However, this is only likely to occur when the anticipated regret intervention is subtle. Blatant anticipated regret interventions are likely to be interpreted as emotional appeals, decreasing the effectiveness of the intervention. Taken together, these studies therefore suggest that people are more likely to undertake an action when they anticipate regret for inaction, and that simply asking people whether they would later regret inaction can significantly increase the likelihood of an action occurring.

### Pilot studies

The aim of the present study is to determine whether simply asking people whether they would later regret not registering as an organ donor increases verified registration. In preparation, we have conducted 3 pilot studies [for full details, see 11,12]. We found that simply asking people whether they would later regret not registering as an organ donor increased their intentions to register [12, Study 2]. Moreover, we replicated this finding with a more representative sample of the adult Scottish general public [12, Study 3]. Although these findings are promising, people do not always act upon their intentions, the well recognised intention-behaviour gap. In a third pilot study we found that asking people whether they would later regret not registering as an organ donor increased self-reported organ donor registration [11]. This latter finding suggests that anticipated regret promotes organ donor registration. However, this research can be criticised for using self-reported measures of registration. The acid test is clearly whether this intervention leads to a significant increase in *verified *registrations on the UK NHS Blood and Transplant (NHSBT) posthumous Organ Donor Register. The aim of the present study is to test whether a large scale, simple anticipated regret intervention leads to a significant increase in NHSBT verified organ donor registrations.

### Research questions

(a) Does a brief, theory-based anticipated regret intervention lead to a significant increase in organ donor registrations?

(b) If we do observe an anticipated regret effect, what is the mechanism, e.g. is it fully mediated via intentions and/or non-cognitive affective attitudes?

(c) What effect size is observed, to inform the power calculation for the next stage, a UK-wide translational study?

(d) What is the feasibility, response rate etc. to inform such a study?

## Method/design

### Recruitment

Participants will be a large, randomly selected representative sample of the adult Scottish general public. These participants will be randomly selected from a list containing 1.2 million members of the adult Scottish general public. Participants will be posted a brief questionnaire, together with information about, and a link to the UK Organ Donor Register. We will be receiving informed consent from all participants. However, we were concerned that if we were to require all participants to return signed consent forms by post, it is likely that people who are more likely to register as an organ donor would be more likely to participate in our study. This would create an unacceptable bias that could render our results scientifically meaningless. We are, therefore, using an 'opt-out' approach in which participants are required to contact us if they do not want to take part in the study. We will make it very clear that people do not have to participate if they do not wish to, and we have made it as easy as possible to withdraw either by; (a) ticking a form and returning it to us in the stamped addressed envelope that we provide to all, (b) email or (c) telephone. We believe that our approach is justified as; (a) no harm will come to the participants, (b) our research cannot be practically carried out if we had to receive written informed consent from all participants, (c) the potential benefits to the NHS (i.e. determining effective methods of increasing organ donor registrations) outweigh the cost, and (d) NHSBT will be conducting the search of the organ donor register, not us, we will never have access to individual identifiable results, and we will never know whether a specific individual has registered or not, only NHSBT will have this information, and crucially, they would have it regardless of whether this study was conducted or not.

Based on our pilot data [11], we estimate that in order to identify a significant effect at the conventional .05 alpha level, at a power of .80, we would need 565 completed questionnaires per condition. As with any questionnaire based study there are likely to be many people who do not complete our survey. The response rate for this type of survey ranges from 23% to 37% [37]. Based on the conservative lower estimate (23%), we calculate that we would need to distribute 2,460 questionnaires per condition in order to gain a sufficient number of completed surveys. Furthermore, we also need to oversample to account for the proportion of the adult Scottish general public that are already registered donors. Adjusting for this, we need to distribute 3,630 surveys per condition in order to gain a sufficient number of completed surveys. Because there are four conditions in this study (for details, see below), we need to distribute a total of 14,520 questionnaires. The logistics of this national field based study will be conducted by Perspektiv, a market research company (http://perspektivred.co.uk). It should be noted that in the present study we improve on O'Carroll et al. [11] by including a no questionnaire control arm. We do not have pilot data that would allow us to conduct power calculations for this condition. However, based on the questionnaire control arm in O'Carroll and colleagues [11], we estimate that the above sample size estimates should be more than sufficient.

#### Exclusion criteria

We are primarily interested in whether a simple anticipated regret intervention increases organ donor registration. We will, therefore, exclude people who are already registered organ donors from the main analyses. We will also exclude participants who actively withdraw from the study.

### Design and materials

**Figure 1:**
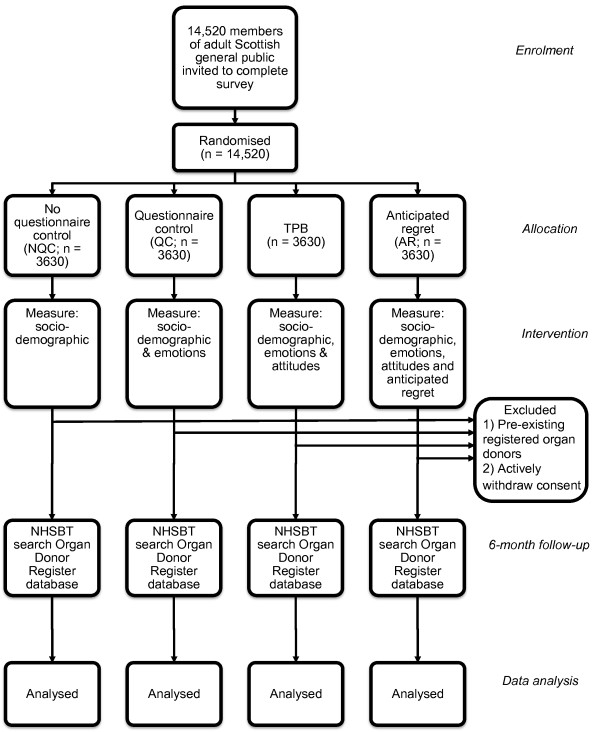
**CONSORT flowchart of trial design**.

We will utilise a between-groups, prospective randomised controlled design (i.e. participants will be randomly allocated into one arm of the study). In line with previous research [11,12,18,34], we will manipulate anticipated regret by altering the questions that the participants complete. Participants will be randomly allocated into one of four conditions: no questionnaire control (NQC), questionnaire control (QC), theory of planned behaviour (TPB) questionnaire, and anticipated regret (AR) questionnaire (for an overview, see Figure 1). The latter three conditions are similar to O'Carroll and colleagues [11].

#### No questionnaire control (NQC)

This extension of O'Carroll and colleagues [11] has been added to determine whether simply being contacted increases organ donor registration. This arm will simply receive a letter, donor registration form and questions collecting demographic information (e.g., date-of-birth, gender, occupation and postcode for socio-economic status estimation).

#### Questionnaire control (QC)

This arm will receive the same materials as the NQC arm plus a questionnaire measuring their emotions and non-cognitive affective attitudes towards organ donation [10-12]. This questionnaire will measure 5 emotions and affective attitudes: the ick and jinx factors, bodily integrity, medical mistrust, and perceived benefit. The ick factor will be measured using three items (e.g. "The thought of organ donation makes me uncomfortable"). Three items will be used to measure jinx (e.g. "The surest way to bring about my own death is to make plans for it like signing an organ donor card"). Two items will measure bodily integrity (e.g. "The body should be kept whole for burial"). There will be four medical mistrust items (e.g. "If I sign an organ donor card, doctors might not try so hard to save my life"). Perceived benefit will be measured with four items (e.g. "Organ donation helps to bring meaning to the death of a loved one"). All items will be rated on a 7-point Likert scale (1 = strongly disagree, 7 = strongly agree).

Non-donors will also be asked to complete two questions measuring their intentions to register as an organ donor in the future (e.g. "I will definitely register as an organ donor in the next few months"; 1 = strongly disagree, 7 = strongly agree). We will also include filler questions to ensure that the number of items in this arm is identical to the TPB and AR arms.

#### Theory of planned behavior (TPB) questionnaire

The TPB arm will complete the same materials as the QC arm, plus additional items measuring attitudes, perceived control, and subjective norms. Attitudes will be measured with two items (e.g. "I support the idea of organ donation for transplantation purposes"). Two subjective norm items will be included (e.g. "Most people who are important to me think I should register as an organ donor in the next few months"). The attitudes and subjective norm items will be rated on 7-point Likert scales (1 = strongly disagree, 7 = strongly agree). Three items will measure perceived control (e.g. "How much control do you have over registering as an organ donor in the next few months?"; 1 = no control, 7 = complete control). Once again, non-donors will also be asked to rate their intentions to register as a donor, using the items described above. We will also include filler questions to ensure that the number of items in this arm is identical to that of the QC and AR arms.

#### Anticipated regret (AR) questionnaire

This arm will complete the same indices as the TPB arm, plus two items measuring anticipated regret: "If I did not register as an organ donor in the next few months I would feel regret" (1 = definitely no, 7 = definitely yes) and "If I did not register as an organ donor in the next few months, I would later wish I had" (1 = strongly disagree, 7 = strongly agree). Non-donors will also be asked to complete the intention indices described above.

#### Organ donor registration

Participants in all four arms of the study will be told how to register as an organ donor online, and by telephone, post and text message. All participants will also receive a NHSBT organ donor registration form.

### Outcomes

Our primary outcome variable is verified organ donor registration within 6 months of our postal intervention. Crucially, NHSBT has agreed to collaborate with us and perform a secure and confidential search of their organ donor register 6 months following our brief postal intervention. This search will tell us whether or not the participant is a registered organ donor and, if applicable, when they registered. NHSBT will conduct this anonymised search for all participants who have not opted out of the study, regardless of whether or not they returned the questionnaire. This will allow us to determine whether there is any bias caused by people being more willing to complete the questionnaire in certain conditions.

Our second outcome is intentions to become an organ donor in the future. If our anticipated regret intervention is successful we will test whether the increase in registration is due to participants having greater intentions to register as an organ donor. This outcome will be measured in our questionnaires, using the indices described above.

### Analyses

Our primary analysis will be a logistic regression predicting donor status (registered vs. not registered) to explore the proportion of respondents who have registered as organ donors 6 months later as a function of the 4 arms. In this analysis we will control for any potential between-arm differences in age, gender and socio-economic status, if these are related to the outcome. We will conduct this regression with all participants who have not opted out of the study, regardless of whether or not they returned the questionnaire. Participants may not comply with their random assignment. That is, in this case, for some reason not complete and return the questionnaire. We will use instrumental variables (IV) regression techniques to estimate the causal effect of the intervention in compliers [see [38,39]]. Here randomization acts as the instrumental variable, compliance status as the endogenous variable. This technique is a viable alternative to traditional intention to treat (ITT) analysis. ITT treats randomization as a treatment, when in actuality it is the intervention, not the randomization that is the source of any effect. By treating assignment as an instrumental variable and intervention compliance as an endogenous variable, IV regression techniques reduce the bias in standard ITT analyses, and provide as estimate of the causal effect of the intervention in compliers [38]. These analyses will be conducted in M*Plus*-6 [40] and Stata [41]. We will also record the time interval between the questionnaire being sent out and date of organ donor registration to test for temporal effects.

If our intervention is successful, we will test the processes through which it occurs. We will assess whether the anticipated regret intervention promotes organ donor registration by increasing people's intentions to register and decreasing their emotions and non-cognitive affective attitudes towards organ donation. Essentially, we will test whether intentions and emotions mediate the effect of our intervention on registration. Simple tests of mediation will be implemented in ZUMASTAT [42]. More detailed tests of multiple and joint mediation as well as moderated mediation will be conducted using M*Plus*.

### Evaluation

The effectiveness of the simple anticipated regret intervention will be assessed using the instrumental variables logistic regression analyses outlined above. This will determine whether there is a significant difference in the proportion of registered organ donors in each arm 6 months following our intervention. If we find that there is greater proportion of participants registered as organ donors in the AR arm than the other three arms, we will conclude that a simple anticipated regret intervention increases organ donor registration. We will also assess the mechanisms behind this intervention. The mediation analyses outlined above will test whether any significant effects of our intervention are due to increases in people's intentions to register and decreases in their emotions and non-cognitive affective attitudes towards organ donation.

### Research ethics and timetable

This study has received ethical approval from the South East Scotland Research Ethics Committee (ref: 11/SS/0093). This is an 18-month project. Months 1-4 will be spent designing, piloting and finalising the layout of the questionnaire pamphlet, and also in working closely with Perspektiv in randomising participants to the four arms of the study. The questionnaire packs will be posted out to participants in months 5 and 6. During months 7-12 the responses will be entered into the PASW spreadsheet as they are returned, and the analytic strategy will be finalised. The protocol for linking the participants to the NHSBT Organ Donor Register will also be developed and finalised during this period. During months 13 & 14 the NHSBT Organ Donor Register will be checked for new registrations by our participants. Months 15-16 will be spent conducting the final analyses, and in months 17-18 we will draft scientific papers, conference presentations and the Chief Scientist Office final report.

## Discussion

A recent report by Nuffield Council on Bioethics [3] highlights the shortage of donor organs and the importance of increasing the number of registered organ donors in order to deal with this shortfall. The UK Government has set aspirational target for increase organ donor registration from its current level of 17 million to 25 million by 2013, and to increase the number of organs donated by 73% [2]. Moreover, with the increase per year in the number of people in the US and UK that are on a waiting list for a solid organ transplant, there is an urgent need to identify and overcome the barriers that deter and to determine facilitators that promote organ donor registration. The aim of this study is to determine whether a simple, theory-based anticipated regret intervention increases NHSBT verified organ donor registration.

Previous research has found that a simple, theory-based anticipated regret manipulation increases people willingness to register as an organ donor [12] and self-reported registration [11]. The aim of the present study is to improve on this research by assessing whether this simple intervention increases *NHSBT verified *organ donor registration. Our proposed intervention is both simple and easy to administer, thereby making it a feasible strategy for improving organ donor registration. If this intervention is successful, we will liaise with our NHS partner (NHSBT) to discuss how this intervention could potentially be used in organ donor campaigns. This research, therefore, has the potential to dramatically increase the number of people who are registered as posthumous organ donors. This will save the lives of people who currently need a transplant and those who may need a transplant in the future. Moreover, with the average cost of dialysis at £30,800 per patient per year [1], this research could potentially save the NHS money by reducing the number of people receiving such treatments.

## Competing interests

The authors declare that they have no competing interests.

## Authors' contributions

REO, EF and PCH obtained a grant to fund this project from the Chief Scientist Office (Scottish Government). LS drafted this protocol paper. REO, EF and PCH critically examined the paper. All authors approved the final manuscript.

## Pre-publication history

The pre-publication history for this paper can be accessed here:

http://www.biomedcentral.com/1471-2458/12/169/prepub
